# Development of a loop-mediated isothermal amplification method for the rapid diagnosis of *Ascochyta rabiei* L. in chickpeas

**DOI:** 10.1038/srep25688

**Published:** 2016-05-10

**Authors:** Xiaolu Chen, Lijuan Ma, Song Qiang, Deying Ma

**Affiliations:** 1 College of Agronomy, Xinjiang Agricultural University, Key Laboratory of the Pest Monitoring and Safety Control of Crops and Forests of the Universities of the Xinjiang Uygur Autonomous Region, Xinjiang Agricultural University, No. 311 Nongda East Road, 830052, Urumqi, Xinjiang, People’s Republic of China

## Abstract

Ascochyta blight (AB) is a devastating fungal disease of chickpeas that has spread to nearly all of the chickpea cultivating regions of the world. The rapid diagnosis of *Ascochyta rabiei* L. (*A. rabiei*), the pathogen that causes AB, plays an important role in *A. rabiei* epidemic tracking and AB management. In this study, a group of loop-mediated isothermal amplification (LAMP) primers was designed to detect *A. rabiei* in chickpea plants and seeds via a LAMP method and a conventional PCR method based on an internal transcribed spacer (ITS) sequence analysis of *A. rabiei*. Compared with the conventional PCR method, the LAMP method not only exhibited greater sensitivity and specificity in the detection of *A. rabiei* but also used simpler equipment and required less operational time. The minimum detectable concentration of the *A. rabiei* genomic DNA solution with the LAMP method was 6.01 × 10^−6 ^ng/μl, which was 100 times lower than that of the conventional PCR method with the same outer primers. The greatest advantage of the LAMP method is that results can be observed via the visualization of color changes in SYBR Green I dye with the naked eye, and it does not require expensive instruments, also with less time consumption.

The chickpea (*Cicer arietinum* L.) is the third-most important food legume around the world, and its area of cultivation is currently approximately 11.5 million ha, which is primarily in developing countries. Ascochyta blight (AB) was first identified and named by Labrousse^1^,^2^. AB is a devastating disease caused by *Ascochyta rabiei* L. (*A. rabiei*) and primarily occurs in chickpea plants. Currently, because of the dispersal of infected seeds and plant debris, AB has spread to nearly all of the main chickpea cultivation regions worldside^3^,^4^,^5^,^6^. In relatively humid and cool weather conditions, AB can develop rapidly, with the initial spore germination occurring on single leaves of the chickpea plant and quickly spreading to surrounding chickpea plants and even to entire chickpea fields. The effects of AB on the chickpea include reduced yields and major degradation of seed quality^7^,^8^. To control the disease, researchers have focused on the pathogenicity of the pathogen and the resistance of chickpea varieties. The relationships between the pathogens’ mating types, pathogenicities and important identification sequences have been clarified^9^,^10^,^11^,^12^,^13^. The diagnosis of infected chickpea seeds and other cultivation components is the first step toward disease management and quarantine inspection, because AB is spread by infected seeds and plant residues. Conventional PCR technology is the primary method used for *A. rabiei* diagnosis. However, this protocol requires a long operation time and expensive equipment, thus hindering monitoring at local quarantine stations and rural plant protection organizations. Thus, a more convenient, rapid and effective method of diagnosing *A. rabiei* is needed.

Loop-mediated isothermal amplification (LAMP) was first reported by Notomi in 2000^14^ and is a sensitive and rapid nucleic acid amplification technology that can amplify nucleic acids under isothermal conditions. The amplification products are stem-loop DNA structures with several inverted repeats of the target fragments or cauliflower-like structures with multiple loops. LAMP is also a highly efficient nucleic acid amplification method that can synthesize large amounts of target nucleic acid fragments in a short time. Consequently, pyrophosphate ions are produced in large amounts and yield white precipitates of magnesium pyrophosphate. On the basis of this white precipitate, it is easy to determine whether the target nucleic acid fragments have been amplified by the LAMP method^14^. Because it has a high efficiency and does not require specialized laboratory facilities, this technique is widely used for the identification of pathogens that affect the health of humans, animals and plants; however, the reported LAMP-diagnosed pathogens have primarily been bacterial^15^,^16^,^17^,^18^ or viral^19^,^20^,^21^, and the utilization of this technique for fungal plant pathogen^22^,^23^,^24^,^25^ detection, particularly *Ascochyta* sp. identification, is rare.

In the present paper, a new and rapid LAMP technology-based method for the detection of *A. rabiei* in chickpea seeds, cultivated soils and plant debris was established and evaluated.

## Results

### Fungi genomic DNA extraction

**Figure 1:**
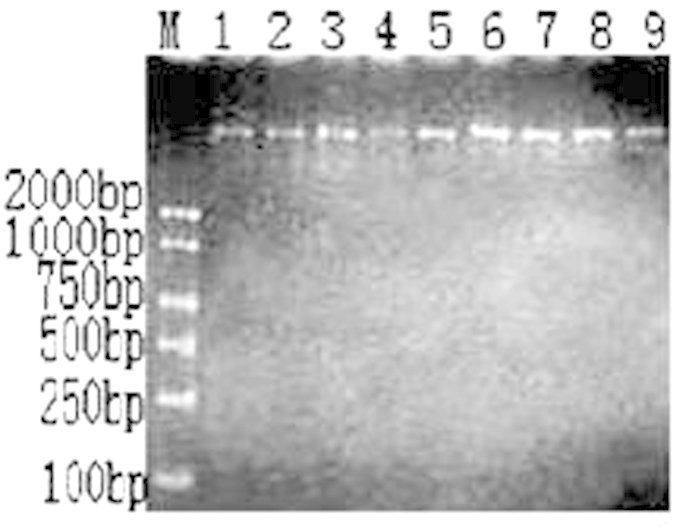
Template genomic DNA samples were extracted by following the protocol of the fungal DNA extraction kit (TIANGEN, Beijing) (M: DL 2000; 1: *A. rabiei*; 2: *Rhizoctonia solani*; 3: *Alternaria alternata*; 4: *Penicillium* sp.; 5: *Aspergillus* sp.; 6: *Nectria* sp.; 7: *Chaetomium* sp.; 8: *Bionectria* sp.; and 9: *F*usarium sp.).

The genomic DNA of *A. rabiei* and 8 other fungi were extracted with fungal genome DNA extraction kits and tested with a NanoDrop 2000 system and electrophoresis. These nine tested fungal genomes were all pure and exhibited light degradation, although their qualities were sufficient for LAMP amplification and conventional PCR reactions (Fig. 1).

### Specificity detection

**Figure 2:**
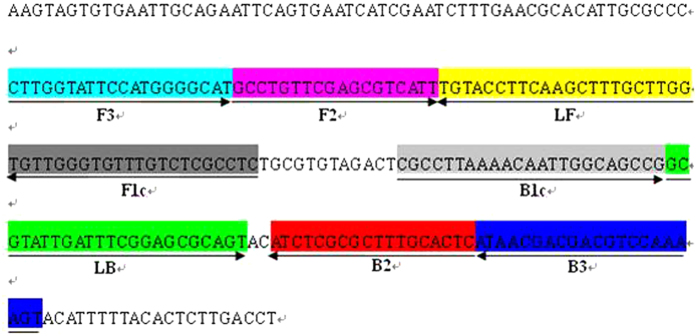
Map of the LAMP primers for *A. rabiei*. The sequence fragment was part of the ITS fragment that was cloned with primers 7-5f/7-5r (unpublished) and considered in the design of the LAMP primers according to a previous study. The FIP and BIP primers contained two distinct regions: F1c plus F2 and B1c plus B2, respectively.

The primers’ specificities were first tested with conventional PCR technology. F3 and B3 (Fig. 2), i.e., the outer primers from the group of LAMP primers, were used in the conventional PCR reactions. In the primer design, the lengths of the fragments amplified by F3 and B3 were 183 bp.

**Figure 3:**
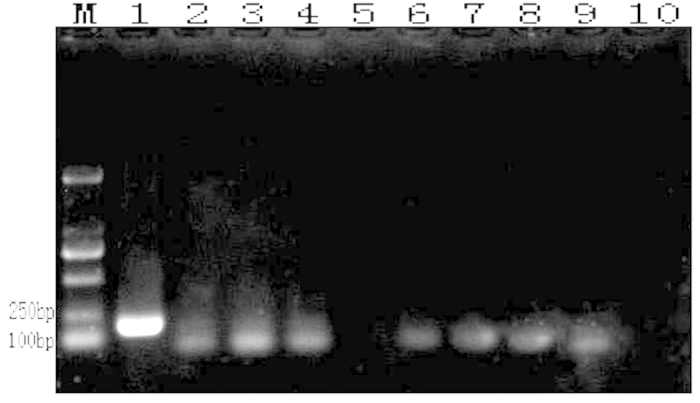
Conventional PCR reaction results (M: DL 2000; 1: *A. rabiei*; 2: *Rhizoctonia solani*; 3: *Alternaria alternate*; 4: *Penicillium* sp.; 5: *Aspergillus* sp.; 6: *Nectria* sp.; 7: *Chaetomium* sp.; 8: *Bionectria* sp.; 9: *Fusarium* sp.; and 10: negative control).

In conventional PCR, the extracted genomic DNAs of *A. rabiei* and the 8 other tested fungal species (details in Table 1) were set as the templates. The amplification products were separated by gel electrophoresis and revealed that only the *A. rabiei* DNA was successfully amplified to yield a fragment of approximately 190 bp, whereas the other 8 fungal PCR amplifications did not yield fragments (Fig. 3).

**Figure 4:**
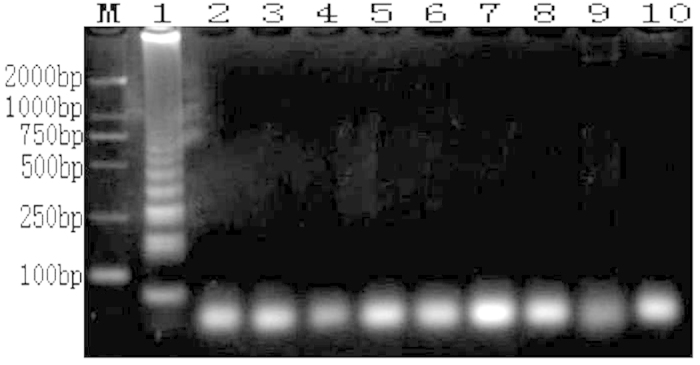
LAMP reaction results tested by electrophoresis (M: DL 2000; 1: *A. rabiei*; 2: *Rhizoctonia solani*; 3: *Alternaria alternate*; 4: *Penicillium* sp.; 5: *Aspergillus* sp.; 6: *Nectria* sp.; 7: *Chaetomium* sp.; 8: *Bionectria* sp.; 9: *Fusarium* sp.; and 10: negative control).

The LAMP reaction products were visualized by electrophoresis on 1% agarose gel. The laddered bands characteristic of a successful LAMP reaction^14^ were present only when the *A. rabiei* template was used, whereas the 8 tested fungal species did not amplify (Fig. 4).

**Figure 5:**
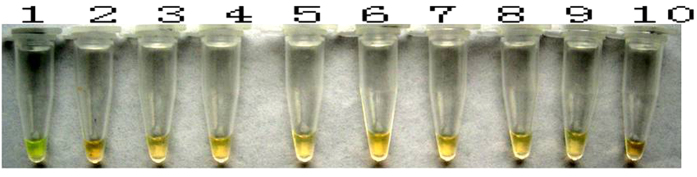
LAMP reaction results visualized with SYBR Green I dye (M: DL 2000; 1: *A. rabiei*; 2: *Rhizoctonia solani*; 3: *Alternaria alternate*; 4: *Penicillium* sp.; 5: *Aspergillus* sp.; 6: *Nectria* sp.; 7: *Chaetomium* sp.; 8: *Bionectria* sp.; 9: *Fusarium* sp.; and 10: negative control).

Thereafter, 1 μl SYBR Green I was added to the 9 LAMP reaction tubes, which were then left to stand for 5 min with light shaking. Only the *A. rabiei* LAMP reaction mixture turned from gold to green, and the colors of the amplification mixtures of the other 8 fungal species did not change. This difference was easily observable with the naked eye. The results are illustrated in Fig. 5.

### Sensitivity detection

**Figure 6:**
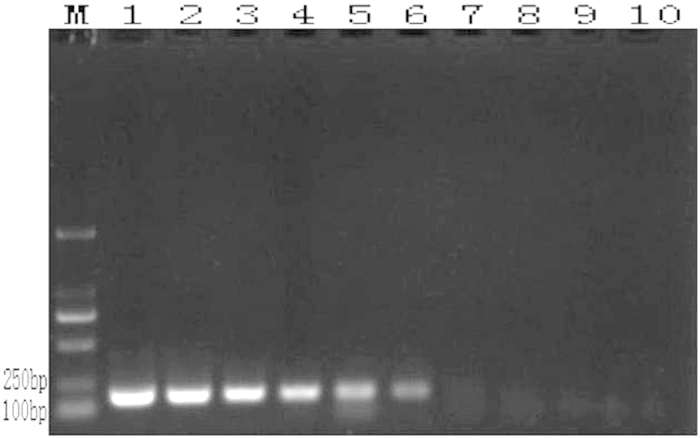
Sensitivity test of conventional PCR with the genomic DNA of *A. rabiei* that had been serially diluted (10 fold) and subjected to 1% agarose gel electrophoresis at 80 V for 30 min and to EB dye and UV light examination (302 nm) (M: DL 2000; template DNA concentration order: 1: 6.01 × 10^1^ ng/μl; 2: 6.01 × 10^0^ ng/μl; 3: 6.01 × 10^−1^ ng/μl; 4: 6.01 × 10^−2^ ng/μl; 5: 6.01 × 10^−3^ ng/μl; 6: 6.01 × 10^−4^ ng/μl: 7: 6.01 × 10^−5^ ng/μl; 8: 6.01 × 10^−6^ ng/μl; 9: 6.01 × 10^−7^ ng/μl; and 10: 6.01 × 10^−8^ ng/μl).

**Figure 7:**
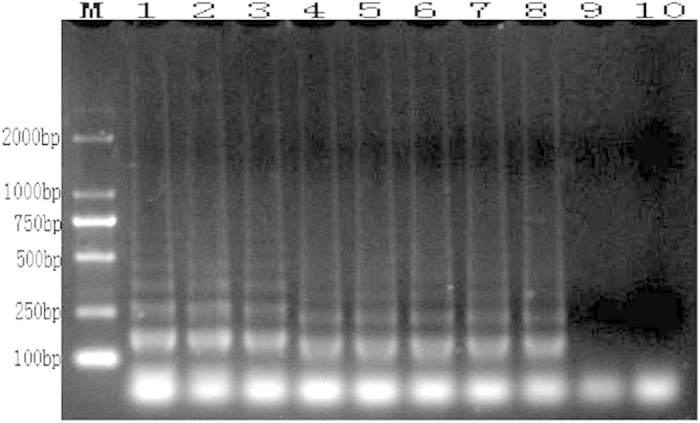
Sensitivity test of LAMP amplification with genome DNA of *A. rabiei* that had been serially diluted (10 fold) (M: DL 2000; template DNA concentration order: 1: 6.01 × 10^1 ^ng/μl; 2: 6.01 × 10^0 ^ng/μl; 3: 6.01 × 10^−1 ^ng/μl; 4: 6.01 × 10^−2 ^ng/μl; 5: 6.01 × 10^−3 ^ng/μl; 6: 6.01×10^−4 ^ng/μl: 7: 6.01 × 10^−5 ^ng/μl; 8: 6.01 × 10^−6 ^ng/μl; 9: 6.01 × 10^−7 ^ng/μl; and 10: 6.01 × 10^−8 ^ng/μl).

**Figure 8:**
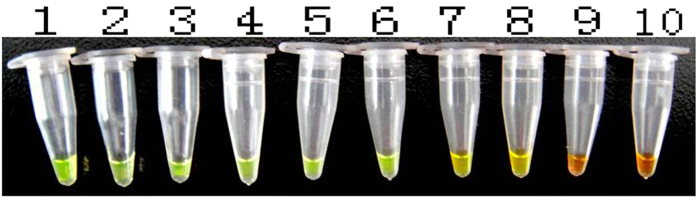
Sensitivity test results for the LAMP amplification as visualized on the basis of color changes in the reaction mixture after the addition of SYBR Green I to the reaction tube (M: DL 2000; template DNA concentration order: 1: 6.01 × 10^1 ^ng/μl; 2: 6.01 × 10^0 ^ng/μl; 3: 6.01 × 10^−1 ^ng/μl; 4: 6.01 × 10^−2 ^ng/μl; 5: 6.01 × 10^−3 ^ng/μl; 6: 6.01 × 10^−4 ^ng/μl: 7: 6.01 × 10^−5 ^ng/μl; 8: 6.01 × 10^−6 ^ng/μl; 9: 6.01 × 10^−7 ^ng/μl; and 10: 6.01 × 10^−8 ^ng/μl).

The original genomic DNA concentration from the fungal genome DNA extract kit was 60.1 ng/μl as evaluated with a NanoDrop 2000. Thereafter, the solution was subjected to 10-fold serial dilution and was used as a template for both conventional PCR and LAMP amplification to measure the sensitivities of these two amplification methods. The reaction products were visualized with 1% agarose gel electrophoresis. With conventional PCR, the minimum template concentration limit at which the target fragments could be amplified and detected via electrophoresis was 6.01 × 10^−4^ ng/μl (Fig. 6). With the LAMP amplification, the minimum template concentration limit at which the target fragments could be amplified and detected via electrophoresis was 6.01 × 10^−6^ ng/μl (Fig. 7). In another detect method that involved the addition of SYBR Green I and visual inspection with the naked eye, when the template concentration was 6.01 × 10^−4^ ng/μl, the color of the LAMP reaction mixture visibly and clearly changed to green. However, when the template concentrations were diluted to 6.01 × 10^−5^ ng/μl and 6.01 × 10^−6^ ng/μl, the colors changed to intermediate colors between gold and green that could not be clearly distinguished (Fig. 8). We estimated that the lowest concentration of the *A. rabiei* genomic DNA that could be clearly identified by discrimination of the color change with the naked eye was 6.01 × 10^−4^ ng/μl in 25 μl in the LAMP amplification system.

### Detection of *A. rabiei* in artificially infected samples

**Figure 9:**
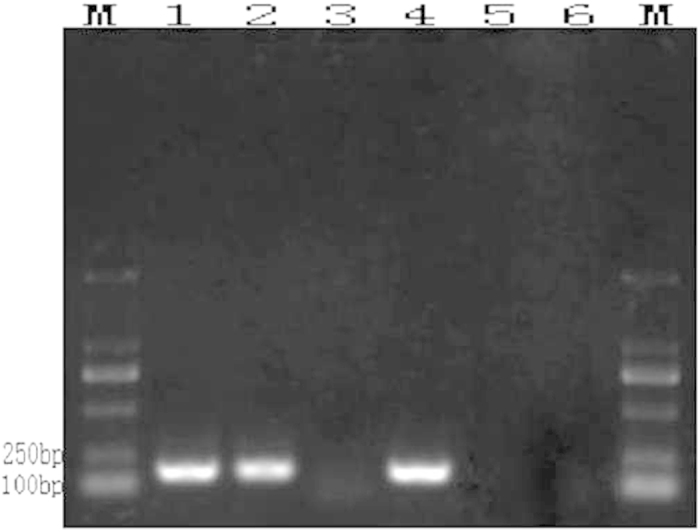
Conventional PCR detection results from the infected chickpeas and soil (M: DL 2000; 1: DNA from diseased chickpea debris; 2: DNA from diseased chickpea seeds; 3: DNA from healthy chickpea seeds; 4: DNA from artificially infected soil; 5: DNA from uninfected sterilized soil; and 6: negative control).

**Figure 10:**
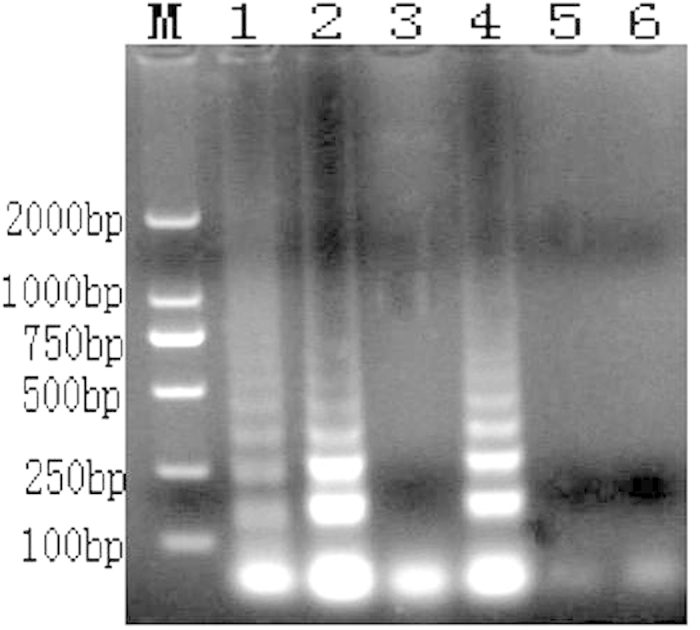
LAMP detection results (M: DL 2000; 1: DNA from diseased chickpea debris; 2: DNA from diseased chickpea seeds; 3: DNA from uninfected chickpea seeds; 4: DNA of soil from diseased chickpea field; 5: DNA from cleaned chickpea field; and 6: negative control).

**Figure 11:**
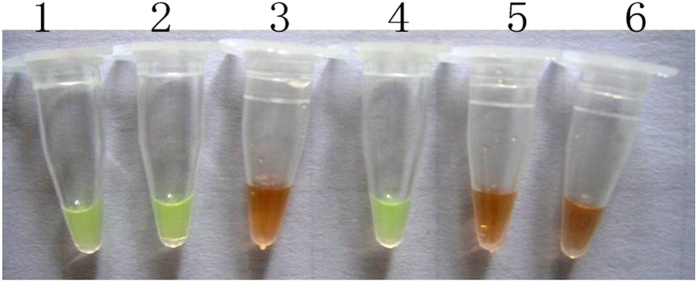
Color changes of the LAMP reaction mixtures with templates from the diseased chickpea plant debris, seeds and soil that had been artificially infected with *A. rabiei* (1: DNA from diseased chickpea debris; 2: DNA from diseased chickpea seeds; 3: DNA from healthy chickpea seeds; 4: DNA from soil from a diseased chickpea field; 5: DNA from a clean chickpea field; and 6: negative control).

With conventional PCR, the approximately 190-bp target fragments of *A. rabiei* obtained from template solutions from diseased chickpea plants, seeds and soil that had been artificially inoculated were all successfully amplified, as visualized with a UV transilluminator. However, the template solutions from healthy chickpea seeds and uninfected sterilized soil did not produce visible results (Fig. 9). Using the LAMP method, we obtained similar results. Electrophoresis on 1% agarose gel yielded only the templates from the diseased chickpea debris, seeds and artificial infected soil, and the LAMP reactions produced the characteristic ladder-pattern bands (Fig. 10). However, the LAMP reaction with the templates from the healthy chickpea seeds and sterilized soil did not produce observable results. On the basis of SYBR Green I staining, the colors of the LAMP reaction mixtures from the diseased chickpea debris, seeds and artificially infected soil changed from gold to green, whereas the colors of the other 3 reaction mixtures did not change (Fig. 11). These amplifications were all performed in three repeats.

## Discussion

The internal transcribed spacer regions (ITS1 and ITS2) of ribosomal DNA have been used in studies to determine the polygenetic relationships of plant pathogens^26^,^27^,^28^ and in many pathogen identification studies^28^,^29^,^30^. Based on these studies, the ITS fragments of *A. rabiei* isolated from Xinjiang Uygur Autonomous Region were cloned and sequenced to improve the rapid diagnosis of *A. rabiei*. In the present study, a novel and high-efficiency protocol based on the LAMP method and conventional PCR for the diagnosis of *A. rabiei* chickpeas was established and evaluated. In the laboratory, the sensitivity of the LAMP amplification was 100-fold greater than that of the conventional PCR method, and the lowest detectable *A. rabiei* genomic DNA solution concentrations detected with a UV transillumination detector were 6.01 × 10^−6 ^ng/μl and 6.01 × 10^−4 ^ng/μl, respectively. When the LAMP amplification results were detected by the naked eye, on the basis of color change of the reaction solution mixed with SYBR Green I, the sensitivity of the LAMP reaction was equal to that of conventional PCR-based conserved fragment amplification as detected with a UV transilluminator, and the minimum detectable genome DNA solution concentrations were 6.01 × 10^−4 ^ng/μl for both methods. The detection sensitivities of the LAMP protocol for the identification of *A. rabiei* in chickpea plants, seeds and artificially infected soil were also evaluated in the laboratory. The results revealed that the diseased chickpea seeds and artificially infected soil could successfully be distinguished from healthy chickpea seeds and sterilized soil through the LAMP amplification method.

Almost all of the locally cultivated chickpea varieties in Xinjiang Uygur Autonomous Region are sensitive or minimally resistant to *A. rabiei*^31^. The breeding of resistant varieties should be an important path to the control of this chickpea disease; however, breeding methods require relatively long time frames. In addition, our previous studies have demonstrated that the strong survival and infection abilities of *A. rabiei* enable it to germinate and infect chickpea plants in moist conditions after preservation for 5 years at room temperature (unpublished observations); thus, total inactivation of *A. rabiei* on chickpea seeds and in cultivation environments for AB management is impractical, and identifying chickpea seeds and field soil that are infected with *A. rabiei* is a more economical method for the management of AB. The rapid and accurate diagnosis of chickpea seeds and cultivation soil may reduce the possibility of AB epidemics and break outs in chickpea cultivation regions. The morphological identification of the characteristics symptoms of AB, i.e., “bulls-eye” lesions of the leaves, circular lesions with gray borders and brown or black inner portions on the chickpea plants and mycelial morphologies^32^, require abundant experience. Pathogen isolation and purification based on cultivation methods require additional time and instrumentation^33^. PCR-based molecular diagnostic methods, such as ITS conserved fragment amplification^26^,^34^,^35^ and cDNA hybridization^36^, also require expensive equipment, skilled operators and time. The advantages of the LAMP method lie in its ability to address these deficiencies: it is less time consuming (approximately 1 h is required to complete the amplification and observations), requires only simple equipment (a water bath and relatively clean experimental conditions) and can be performed by people without extensive training.

LAMP’s favorable characteristics give LAMP the potential to provide rural plant protection organizations and basic-level agriculture governments with a rapid method to detect AB and infected chickpea seeds/plants and hence prevent outbreaks. These advantages of the LAMP method may improve *A. rabiei* quarantine inspection efficiency in intra- and international chickpea seed trade systems and chickpea germplasm introduction systems. Thus, the potential for AB epidemics and breakouts in chickpea cultivation regions may be reduced or managed from the source of the pathogen. Moreover, because the apparatus requirements of the LAMP method are practical, the operating time is minimal, and the operation steps are simple, the LAMP method could be used in many circumstances for the diagnosis of *A. rabiei* in chickpea plants, seeds and soil from the field, and this may help chickpea cultivators to enhance the management of AB and reduce the economic losses associated with *A. rabiei* in chickpeas.

## Materials and Methods

### Materials

#### Tested fungal strains

1. *A. rabiei* (mating type 1-1, unpublished data) samples were isolated from chickpea debris that was spontaneously infected with AB in an open field. Stems and pods with typical symptoms were collected from Mulei County and cut into 3–4 mm pieces after the surfaces were disinfested and cleansed with a 2% sodium hypochlorite solution for 1.5 min. Subsequently the samples were washed with sterile distilled water (SDW) 3 times and plated on ¼ strength EPDA medium (chickpea and potato dextrose agar: chickpea seed meal 50 g; potato 100 g; dextrose 20 g; agar 20 g; distilled water 1 L). The plated samples were incubated for 10 days in artificial cultivation chambers (SPX-300I-G, Boxun Co., Ltd., Shanghai) at 25 °C on an alternating 12-h light-dark cycle as reported by Kaiser *et al.*^37^,^38^,^39^. To acquire the purified pathogen fungi, single spores were cultivated on EPDA medium under the same conditions.
2. The following 9 common plant pathogens or fungal species were used in the laboratory experiment: *Rhizoctonia solani*, *Alternaria alternate*, *Penicillium* sp., *Aspergillus* sp., *Nectria* sp., *Chaetomium* sp., *Bionectria* sp., *and Fusarium* sp. The samples had been previously purified and stored at Xinjiang Agriculture University and were identified to the genus level through morphological and molecular methods. In the present study, these fungal strains were all re-cultivated on PDA medium under the same cultivation conditions as *A. rabiei* to generate sufficient mycelia and spores for genome extraction. Detailed information on these fungal species is displayed in Table 1.
3. Diseased chickpea debris and artificial infected soil samples. The diseased chickpea debris was collected from Mulei County, which is the most substantial chickpea cultivation region in China. The debris was collected in open fields with diseased chickpeas exhibiting typical AB symptoms. These debris samples were sealed in plastic bag after being naturally dried and stored at room temperature. The artificially infected soil samples were collected from a chickpea cultivation field, naturally dried and then sterilized at 120 °C for 30 min, and then dried again as previously described. With the exception of the negative control, 10 g sterilized treated soil samples were mixed with 1 g of the mycelia or spores of the cultivated *A. rabiei* to create the fungus-infected soil samples. These artificially infected soil samples and the uninfected soil samples were all ground with a mini-grinder separately and then stored at 4 °C. All of the artificially infected and blank soil samples were examined in 3 biological replicates in this study.

### Reagents and instruments

The following reagents and instruments (with their suppliers) were used: fungal genome DNA extraction kit (TIANGEN, Beijing), Bst DNA polymerase (New England Biolabs, Beijing), betaine (Sigma Aldrich, Shanghai), agarose (BIOWEST, Hong Kong), Taq DNA polymerase (Takara, Japan), dNTPs (Beijing Zoman Biotechnology Co., Ltd., Beijing), Marker-DL2000 (Beijing Zoman Biotechnology Co., Ltd., Beijing), SYBR Green I (Biotech Co., Ltd., Beijing), isothermal water bath kettle DK-8D (Beijing Jinyi Tech. Co., Ltd., Beijing), PCR-Cycler (Biometra, Germany), electrophoresis apparatus DYY26C (Beijing Liuyi Co., Ltd., Beijing), Bio-Rad multimager (Richmond, CA), and NanoDrop 2000 (Thermo Fisher, USA).

## Methods

### DNA template extraction

Following the protocol of the fungal genome DNA extraction kit (TIANGEN, Beijing), the genomic DNAs of the 9 tested fungal strains that had been cultivated on the media were extracted, quality tested with a NanoDrop 2000, subjected to 1% agarose gel electrophoresis, dyed with EB, examined under UV light (280 nm), and stored at −20 °C.

The genomic DNA extracts of the fungi from the diseased chickpea debris and artificially infected soil samples were subjected to different protocols. The diseased plant samples from the diseased chickpea debris were frozen with liquid nitrogen, finely ground with a mortar and pestle, and then subjected to the protocol of the fungal genome DNA extraction kit (TIANGEN, Beijing). Furthermore, after quality testing with a NanoDrop 2000 and 1% agarose gel electrophoresis, the samples were dyed with EB and examined under UV light (280 nm) and subsequently stored at −20 °C. The DNA solutions from the artificial infected soil samples were extracted via the CTAB method^40^ with slight modifications. Briefly, each 0.1 g soil sample was carefully ground with 1 mL lysis buffer (Tris-HCl 100 mM, pH = 9.0, EDTA 100 mM, NaCl 400 mM, 2% SDS) and 0.5 g of quartz sand in a mortar. Next, the solution (approximately 500 μL) was transferred to a 1.5 mL centrifuge tube and incubated for 1 h at 65 °C with shaking every 10 min. After incubation, the samples were centrifuged for 10 min at 12,000 rpm. The upper liquid was retained, sequentially extracted with an equal volume of a phenol:chloroform:isoamylol mixture (24:24:1 by volume), and centrifuged for 10 min at 12,000 rpm. The supernatant was transferred to a fresh 1.5 mL microtube, the same volume of chloroform was added, and the microtube was shaken gently for 5 min and subsequently centrifuged for 10 min at 12,000 rpm. The supernatant was transferred to a fresh microtube, precipitated with a 3/4 volume of isopropanol, washed twice with 75% ethanol, dried, and redissolved in 200 μL TE buffer (1 mM Tris-HCl, 0.1 M EDTA, pH = 8.0). Two microliters of RNase (10 mg/mL) was added, and the sample was subjected to testing with a NanoDrop 2000 and 1% agarose gel electrophoresis at 80 V for 30 min, dyed with EB, examined under UV light (280 nm), and then stored at −20 °C.

### Primer design and synthesis

The sequence information for the ITS region^41^ of *A. rabiei* that had been analyzed in a previous study and documented in GenBank (accession number: JF714463) was collected and BLAST searched on NCBI with other sibling species to identify the conserved region. The conventional PCR primers 7-5f/7-5r were designed with Primer Premier 6.11 DEMO (PREMIER Biosoft). Then, the cloning and sequencing of the target fragments from *A. rabiei* that were isolated from the infected chickpea debris from the open field in Xinjiang were completed. On the basis of the *A. rabiei* ITS sequence information and an online LAMP primer design software (www.primerexplorer.jp), one group of outer primers, i.e., F3 and B3, one group of inner primers, i.e., FIP and BIP, and one pair of loop primers, i.e., LF and LB, were determined and synthesized by BGI Co., Ltd. Information on these primers is presented in Table 2 and Fig. 2.

### Amplification reaction system and program

#### Conventional PCR

F3 and B3 were first set as the amplification primers for the conventional PCR to demonstrate the specificity of this group of LAMP primers. The PCR reaction system contents were as follows:, 2.5 μl 10 × buffer; 0.5 μl dNTP (10 mM each); 0.5 μl (10 μM each) F3 and B3; 0.5 μl Taq DNA polymerase; 6 ng DNA template, and ddH_2_O to a total volume of 25 μl. The negative control was set using ddH_2_O as the template. The following thermal cycle parameters were used: 30 cycles of predenaturing at 94 °C for 4 min, denaturing at 94 °C for 1 min, renaturing at 54 °C for 30 s, elongation at 72 °C for 30 s, final elongation at 72 °C for 10 min, and storage at 4 °C. The PCR products were tested via 1% agarose gel electrophoresis at 80 V for 30 min, dyed with EB and examined under UV light (302 nm).

#### LAMP reaction

The reaction system contained 1.6 μM each of FIB and BIP, 0.2 μM each of F3 and B3, 0.8 μM each of LF and LB, 10 mM each of the dNTPs, 2.5 μl 10 × LAMP buffer (KCl, MgSO_4_, (NH_4_)_2_SO_4_, Tris–HCl, TritonX-100), 2 μl MgSO_4_, 8 U of the BstDNA polymerase large fragment, 6 ng of the DNA templates, and ddH_2_O to a total volume of 25 μl. For the negative control, ddH_2_O was used as the template. The following LAMP amplification program was used: 65 °C incubation for 45 min followed by 80 °C incubation for 10 min to stop the reaction. Three methods were used to investigate the LAMP reaction results: the LAMP reaction tube was centrifuged to investigate the white sediment with the naked eye, the LAMP reaction products were detected per the conventional PCR product detection protocol, and the LAMP reaction products were detected with SYBR Green I staining, which involved addition of 1 μl SYBR Green I to each LAMP reaction tube followed investigation of the color change by the naked eye.

These two types of amplification systems and programs were all examined in 3 repeats.

#### Specificity test

The specificities were tested via both the LAMP and PCR methods. The amplification template was substituted with ddH_2_O for the blank control, and genomic DNA solutions of the 9 cultivated fungal species were used as the amplification templates in 3 repeats of both the LAMP and conventional PCR methods. Genomic extractions from the diseased chickpea debris and the artificially infested soil were also used for specificity testing. The PCR amplification products were detected with electrophoresis on a 1% agarose gel at 80 V for 30 min and visualized under UV light, and the LAMP products were investigated with the naked eye for color changes after the addition of 1 μl SYBR Green I to each LAMP reaction tube.

#### Sensitivity analysis

The sensitivities of the LAMP and conventional PCR methods were compared by using 10–fold serial dilutions of the *A. rabiei* genomic DNA solution, which resulted in concentrations ranging from 6.01 × 10^−1 ^ng/μl to 6.01 × 10^−8 ^ng/μl. Thereafter, we investigated the amplified products with the different methods as previous described.

## Additional Information

**How to cite this article**: Chen, X. *et al.* Development of a loop-mediated isothermal amplification method for rapid diagnosis of *Ascochyta rabiei* L. in chickpeas. *Sci. Rep.* **6**, 25688; doi: 10.1038/srep25688 (2016).

## Figures and Tables

**Table 1:** Details of the tested fungal strains.

Tested NO.	Isolates	Hosts or isolated	Location
1	*Ascochyta rabiei* L.	Chickpea	Mulei County, Xinjiang
2	*Rhizoctonia solani*	Cotton	Urumqi, Xinjiang
3	*Alternaria alternata*	Cucumber	Urumqi, Xinjiang
4	*Penicillium* sp.	Soil	Urumqi, Xinjiang
5	*Aspergillus* sp.	Soil	Urumqi, Xinjiang
6	*Nectria* sp.	Cotton	Urumqi, Xinjiang
7	*Chaetomium* sp.	Soil	Urumqi, Xinjiang
8	*Bionectria* sp.	Soil	Urumqi, Xinjiang
9	*Fusarium* sp.	Cotton	Urumqi, Xinjiang

**Table 2:** Primer sequence information for the LAMP reaction.

Label	Len	Tm (°C)	5′dG	3′dG	G/C	Sequence(5′ → 3′)
F3	20	59.53	−5.00	−6.24	0.50	CTTGGTATTCCATGGGGCAT
B3	21	59.49	−3.66	−4.06	0.43	ACTTTTGGACGTCGTCGTTAT
FIP	40					GAGGCGAGACAAACACCCAACA-GCCTGTTCGAGCGTCATT
BIP	41					CGCCTTAAAACAATTGGCAGCCG-GAGTGCAAAGCGCGAGAT
LF	22	61.06	−5.85	−4.74	0.45	CCAAGCAAAGCTTGAAGGTACA
LB	23	65.65	−5.30	−6.57	0.52	GCGTATTGATTTCGGAGCGCAGT

The *A. rabiei* target amplification fragment length using the outer primers F3 with B3 was 183 bp.
